# Extracellular Matrix Protein Signatures of the Outer and Inner Zones of the Rat Adrenal Cortex

**DOI:** 10.1021/acs.jproteome.4c00071

**Published:** 2024-07-17

**Authors:** Jean Lucas Kremer, Veronica Feijoli Santiago, Fernanda Bongiovani Rodrigues, Thais Barabba Auricino, Danilo Henriques de Oliveira Freitas, Giuseppe Palmisano, Claudimara Ferini Pacicco Lotfi

**Affiliations:** † Institute of Biomedical Sciences, Department of Anatomy, University of São Paulo, Av. Prof. Lineu Prestes, 2415, Butantã, São Paulo, SP 05508-000, Brazil; ‡ Institute of Biomedical Sciences, Department of Parasitology, University of São Paulo, Av. Prof. Lineu Prestes, 1374, Butantã, São Paulo, SP 05508-000, Brazil

**Keywords:** extracellular matrix, adrenal gland, adrenal cortex, rat

## Abstract

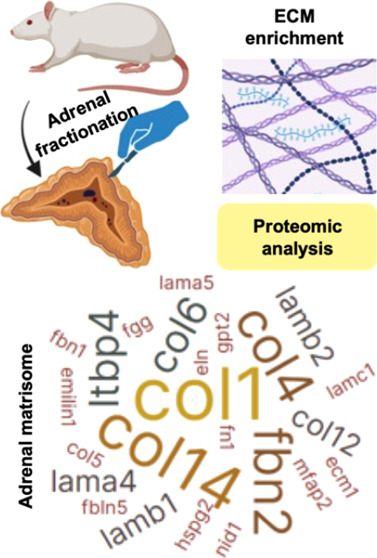

This study analyzes the extracellular matrix (ECM) signatures of the outer (OF = capsule + subcapsular + zona glomerulosa cells) and inner fractions (IF = zona fasciculata cells) of the rat adrenal cortex, which comprise two distinct microenvironment niches. Proteomic profiles of decellularized OF and IF samples, male and female rats, identified 252 proteins, with 32 classified as ECM-component and ECM-related. Among these, 25 proteins were differentially regulated: 17 more abundant in OF, including Col1a1, Col1a2, Col6a1, Col6a2, Col6a3, Col12a1, Col14a1, Lama5, Lamb2, Lamc1, Eln, Emilin, Fbln5, Fbn1, Fbn2, Nid1, and Ltbp4, and eight more abundant in IF, including Col4a1, Col4a2, Lama2, Lama4, Lamb1, Fn1, Hspg2, and Ecm1. Eln, Tnc, and Nid2 were abundant in the female OF, while Lama2, Lama5, Lamb2, and Lamc1 were more abundant in the male IF. The complex protein signature of the OF suggests areas of tissue stress, stiffness, and regulatory proteins for growth factor signaling. The higher concentrations of Col4a1 and Col4a2 and their role in steroidogenesis should be further investigated in IF. These findings could significantly enhance our understanding of adrenal cortex functionality and its implications for human health and disease. Key findings were validated, and data are available in ProteomeXchange (PXD046828).

## Introduction

The adrenal gland comprises two regions: the cortex and the medulla, which have distinct embryological origins, mesoderm and neuroectoderm, respectively. The adrenal cortex is part of the hypothalamic-pituitary-adrenal axis and is characterized by its unique histological architecture, comprising distinct zones that exhibit differential steroidogenic functions: the zona glomerulosa (zG), zona fasciculata (zF), and the zona reticularis (zR), responsible for producing glucocorticoids, mineralocorticoids, and androgens, respectively, although in murine the zR is not recognized.^1,2^ The functional heterogeneity of these zones arises from the complex interplay between endocrine and paracrine signaling mechanisms, as well as the intricate and little-understood organization and composition of the extracellular matrix (ECM).^3^ The ECM, a dynamic network of macromolecules surrounding cells within tissues, not only provides structural support but also actively participates in regulating the cellular microenvironment and tissue homeostasis.^4^ The composition and organization of the ECM are crucial for proper tissue function, with abnormalities in ECM components or their interactions potentially leading to pathological conditions.^5^ While the role of the ECM in various organs has been extensively studied,^6,7^ our understanding of the composition and function of the ECM within the adrenal cortex remains limited. Studies of the ECM in rat and bovine adrenal cortexes have described its potential influence on adrenocortical steroidogenesis, cell proliferation, and tissue remodeling.^8^ Several key rat adrenal cortex ECM components, such as collagens, particularly types 1, 2, and 4, have been detected in the interstitial spaces between adrenocortical cells and around blood vessels.^9^ Fibronectin, an adhesive glycoprotein, is also present in the rat adrenal ECM, likely playing a role in cell-matrix interactions.^10^ Matricellular proteins, such as thrombospondins, have been identified and implicated in cell adhesion and tissue remodeling processes.^10^ Additionally, proteoglycans, including heparan sulfate proteoglycans, and other ECM proteins such as laminins have been observed within the rat adrenal ECM, potentially contributing to the regulation of growth factors and cellular signaling.^11−13^ However, the precise composition, spatial distribution, and functional relevance of these ECM components in the adrenal cortex remain to be fully elucidated. This work aims to comprehensively identify the constitution of the ECM of the rat adrenal cortex, focusing on specific extracellular matrix components of the outer and inner zones and their possible roles in regulating adrenocortical structure and function. Aiming to identify the ECM signature of outer (OF) and inner fractions (IF) obtained from dissected rat adrenal glands, we compared the proteomic profiles of decellularized fractions between OF and IF, and between male and female OF and IF samples. We validated key findings by using histochemistry and immunohistochemistry and the relevance of the observed differentially expressed ECM and ECM-related protein signatures for adrenal structure and function. Finally, a better understanding of the ECM constitution of the adrenal cortex could provide valuable information about the regulation of adrenal gland function, maintenance, and renewal.

## Experimental Section

### Animals

Male and female Sprague–Dawley adult rats, with an average weight of 250 ± 30 g, were obtained from the General Animal House of the “City Hall” of the University of São Paulo Campus of Ribeirão Preto (PUSP-RP) and maintained in a temperature-controlled environment and 12 h light/dark cycle. The Animal Experimentation Ethics Committee from the Biomedical Sciences Institute approved the study (CEUA *n*° 4404220321). The animals were fed standard rat feed and received water ad libitum. All experimental procedures were conducted between 09 and 11 am. Rats were euthanized by decapitation, and their adrenal glands were harvested.

### Outer and Inner Fractions from Rat Adrenal Samples

The preparation of rat adrenal samples was described by Mattos and Lotfi, and Mendonca.^14,15^ The adrenal glands were removed and carefully decapsulated to separate the capsule, subcapsule cells, and zona glomerulosa (outer fraction/OF) from the zona fasciculata, “reticular” and medulla. The medulla was separated from the zona fasciculata and “reticular” to obtain the inner adrenal (IF). Six OF and IF samples were obtained from the rat adrenal glands. Three samples of each were from male and female adrenal glands, respectively. Each sample represents a pool of five animals, totaling 10 glands. After obtaining the fraction samples, they were frozen at −80 °C for at least 24 h before decellularization.

### Preparation of Adrenal-Derived ECM

ECM enrichment through decellularization was obtained through a four-step process. The adrenal fractions were incubated in 1% Triton-X (Sigma-Aldrich, St Louis, MO; 10 mL/cm^3^) for 2 h 30 min at room temperature (RT) under mild agitation, followed by incubation in 4.0% sodium deoxycholate (Sigma-Aldrich) for 3 h. Afterward, the samples were incubated at 4 °C in 1.0% sodium deoxycholate overnight and for another 3 h at RT in 4.0% sodium deoxycholate. All of the steps were followed by four 15 min washes with sterile water. The material was stored at −80 °C. The remaining intact cells were certified by DNA quantification in NanoDrop (Thermo Fisher Scientific) and considered adequate at a concentration <50 ng/μL.^16^ (Figure S1A). Decellularized samples of adrenal and intact tissue (control) were stained with 4 mg/mL 4′,6-diamidino-2-phenylindole-DAPI for 5 min and analyzed under a fluorescence microscope (Nikon; Figure S1B).

### Sample Preparation for Proteomic Analysis

Pellets containing ECM proteins were resuspended in 1% sodium deoxycholate in 1 mM DTT and incubated for 30 min at 56 °C until the matrix was completely solubilized. Samples were then sonicated 3 times for 30 s each, using a probe tip sonicator on ice at 40% power. Extracted proteins were reduced with 10 mM DTT for 30 min at 56 °C and subsequently alkylated in 40 mM iodoacetamide for 30 min in the dark. Following incubation, the samples were digested with trypsin 2% (w/w) overnight at 37 °C. The samples were acidified with 1% trifluoroacetic acid and centrifuged at 10,000*g* for 10 min to stop trypsin digestion and remove insoluble material (e.g., lipids). The supernatant was collected and desalted using self-made microcolumns made with a C18 plug taken from a C18 disk (Sigma-Aldrich) and inserted in the constricted end of a P200 tip. The acidified samples were loaded into the C18 disk (Sigma-Aldrich) microcolumn by applying gentle air pressure with the syringe and washed 3 times with 0.1% TFA. Peptides were eluted with 50% acetonitrile (ACN), 0.1% TFA, and 70% ACN, 0.1% TFA. Digested peptides were dried in a speed vacuum concentrator.

### Liquid Chromatography Coupled to Mass Spectrometry Analysis

Peptide samples were resuspended in 0.1% formic acid (FA) and analyzed using an EASY-nLC system (Thermo Fisher Scientific, Waltham, MA) coupled to an LTQ-Orbitrap Velos mass spectrometer (Thermo Fisher Scientific). Peptides were loaded on Reprosil-Pur C18-AQ (3 μm) column and separated in a gradient from 100% phase A (0.1% FA) to 2–30% phase B (0.1% FA, 95% ACN) for 80 min, followed by 30–95% B for 5 min and 95% of B for 20 min at a constant flow rate of 300 nL/min. LTQ-Orbitrap Velos instrument was operated in positive ion mode with data-dependent acquisition. Each MS scan was acquired at a resolution of 60,000 fwhm followed by 21 MS/MS scans of the most intense ions. Peptides were fragmented by collision-induced dissociation (CID) with a normalized collision energy of 35 and an activation time of 10 ms. Ions selected for MS/MS were dynamically excluded for 30 s. All raw data were accessed using Xcalibur software (Thermo Fisher Scientific).

### Data and Bioinformatics Analysis

**Figure 1:**
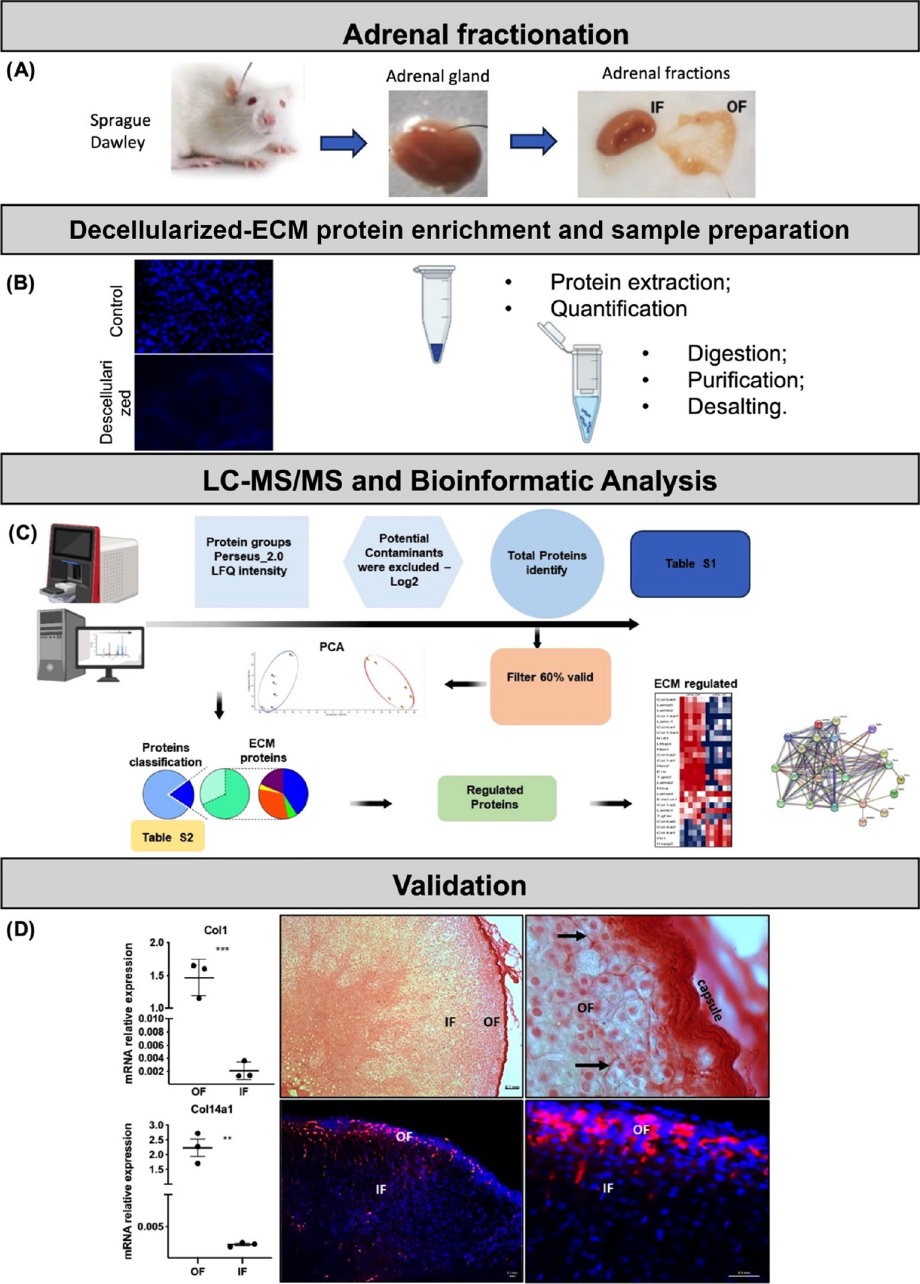
Schematic overview of the conceptual methodology of the different study phases. (A) Collection and fractionation of Sprague–Dawley rat adrenal glands. Obtaining external (OF) and internal (IF) fractions from an adrenal pool for each sample; *n* = 6 samples. (B) Decellularization for the extracellular matrix (ECM) enrichment and sample preparation for proteomic analysis. DAPI staining certified the absence of the remaining intact cells. (C) ECM protein quantification through a high-resolution proteome-wide quantification LC–MS/MS and bioinformatic analysis. (D) Validation by immunohistochemistry of proteins differentially expressed identified in OF and IF samples.

Raw liquid chromatography with tandem mass spectrometry (LC-MS/MS) files were processed using MaxQuant software version 2.1.4 employing the Andromeda search engine on SwissProt *Ratus norvegicus* and *Mus musculus* combined databases (54,778 and 17,121 entries, respectively, downloaded from Uniprot.org November 2022). Database searches were performed with the following parameters: precursor mass tolerance of 10 ppm, product ion mass tolerance of 0.6 Da; trypsin cleavage with two missed cleavages allowed; carbamidomethylation of cysteine (57.021 Da) was set as a fixed modification; and oxidation of methionine (15.994 Da) and protein N-terminal acetylation (42.010 Da) were selected as variable modifications. All identifications were filtered to achieve a protein peptide and PSMs, a false discovery rate (FDR) of less than 1%, and a minimum of one unique peptide was required for protein identification. Moreover, for protein quantification, at least two unique peptides were considered for each protein. Potential common contaminants, proteins identified in the decoy database, and proteins only identified by site were excluded. Protein quantification was based on the MaxQuant label-free algorithm, using both unique and razor peptides for protein quantification. Protein abundance was assessed on label-free protein quantification (LFQ) based on the extracted ion chromatogram area of the precursor ions, activating matching between run features. Statistical analysis was performed using Perseus software (https://maxquant.net/perseus/). Based on LFQ intensity, the data was log2 transformed, assuming a minimum of 60% of valid values in at least one group. The 60% filter of missing values was applied to increase the confidence of the results, considering that in at least one group, 60% of the samples (a minimum of three out of five samples) should have valid quantitative values. A Student’s *t* test (*p* < 0.05) was applied to identify the significant proteins between OFs and IFs. The total identified and regulated proteins were compared with MatrisomeDB 2.0 (https://matrisomedb.org/), (doi.org/10.1093/nar/gkz849). The proteins were annotated using UniProtKB codes (https://www.uniprot.org/) and Gene Ontology AmiGO2 (https://amigo.geneontology.org/amigo/landing). Protein–protein interaction networks and functional enrichment analysis were performed using the STRING platform (https://string-db.org/). A visually effective summary was generated using the BioRender software (https://www.biorender.com/). The workflow of proteomic analysis is presented in Figure 1.

### qPCR

Total RNA was extracted using Trizol (Invitrogen) as described by the manufacturer. RNA integrity and its concentration were evaluated by agarose gel electrophoresis (2%) and spectrometry (NanoDrop 2000c, Thermo Fisher Scientific, Waltham, MA). The cDNA was generated from 4 μg of RNA using M-MLV Reverse Transcriptase (Invitrogen). The quantitative real-time reverse-transcription polymerase chain reaction (qRT-PCR) was performed using the 7500 Real-Time PCR System Sequencer (Applied Biosystems, Foster City, CA) and SybrGreen reagents (Applied Biosystems) and primers (Table 1).

**Table 1:** Sequence of Primers Used in the qRT-PCR Assay^a^

gene	forward	reverse
Col1a1	CAGGCTGGTGTGAGTGGGATT	AAACCTCTCTCGCCTCTTGC
Col14a1	CTGTGCTGGATGACGGAAGT	GGAGAACTGAAGTCACGGGG
Fbn2	GCAGAAATACCCCTGGCAGT	GACACACGGGTTGCTTTCAC
Col4a1	TGCCAAGCACGGAAGAGAATG	GTACCCAGCTATTAAGGCTGCT
Nid-2	GACACTAGTCACAGCAGGGG	TTGTAGGACTCTTTGGGGCG
β actin	TGGTGATGGAGGAGGTTTAGTAAGT	AACCAATAAAACCTACTCCTCCCTTAA
GusB	GACACGCTAGAGCATGAGGG	TCTGCATAGGGGTAGTGGCT

a Col1a1—Collagen type I α 1; Col14a1—collagen type XIV α 1; Fbn2—fibrillin 2; Col4a1—collagen type IV α 1; Nid2—nidogen-2; βactin—β-actin; GusB—glucuronidase β.

A cycle threshold (CT) value was selected in the linear range of amplification for each sample in triplicate and was normalized by endogenous control genes GUSB (human β-glucuronidase) and/or β-actin. The relative expression levels were calculated using the 2^–ΔΔCt^ method.^17^ The data from three different experiments were presented as mean ± SD.

### Histochemistry and Immunohistochemistry

Rat adrenal glands were dissected from adherent adipose tissue, fixed in 4% paraformaldehyde (Merck, Darmstadt, Germany) dehydrated, and embedded in paraffin. Histological sections of 8 μm were performed using a conventional microtome. For histochemistry staining of collagen type 1 (Col1), the sections were incubated in a 0.1% Sirius Red solution in aqueous picric acid solution for 40 min, washed in water, and mounted in a Permount mounting medium (Fisher Chemical). The sections were analyzed under a light microscope (Nikon) using Microlucida Explorer software (MBF Bioscience, VT). For immunofluorescence detection, sections were immersed in citrate buffer (pH 6.0) at 96 °C for 20 min, followed by washing in phosphate buffer solution (PBS) with 0.025% of Triton (PBST) for antigen retrieval. The sections were blocked with antigoat serum for 1 h and further incubated at 4 °C overnight with antibody anticollagen XIV α 1 [Col14α1 (1:100), Invitrogen, PA5–68472] and anticollagen IV [Col4 (1:100), Abcam, ab-6586], and antilama2 [Lama2 (1:250), Santa Cruz, sc-H130]. Secondary antibody Alexa Fluor 594 antirabbit IgG fluorescent antibody (Jackson ImmunoResearch Laboratories; 1:2000, PA) was used for 1 h followed by DAPI (4′,6-diamidino-2-phenylindole; Sigma-Aldrich; MA) for nuclear staining. The sections were mounted in VECTASHIELD PLUS Antifade Media (H-1900, Vector Laboratories Burlingame, CA) and analyzed with a fluorescence microscope using ZEN lite. Software (Zeiss Microscopy). The antifibrillin 2 [Fbn2 (1:200), Bioss-Antibodies, bs-12166R] was detected by immunoperoxidase staining using Vectastain Elite ABC kit according to the datasheet (PK-6101, Vector Laboratories, Burlingame, CA) and diaminobenzidine (DAB, Sigma, D8001). The anti-Fbn2 was analyzed under a light microscope (Nikon) and Microlucida Explorer software (MBF Bioscience, VT). The adrenal sections incubated in nonimmune primary sera tested negative (data not shown).

### Statistical Analysis

Statistical analysis was performed using the GraphPad Prism 9 program, and the results were considered mean ± SD. Results were considered significant for *p* < 0.05.

## Results and Discussion

### Quantitative Proteomics Applied to ECM Derived from the Outer and Inner Fractions of Rat Adrenal Cortex

ECM from different fractions of rat adrenal glands was obtained through a specific decellularization protocol. A workflow was applied for the extracted ECM from outer and inner fractions of rat adrenal glands using label-free quantitative LC–MS/MS-based proteomics combined with validation by immunohistochemistry (Figure 1).

**Figure 2:**
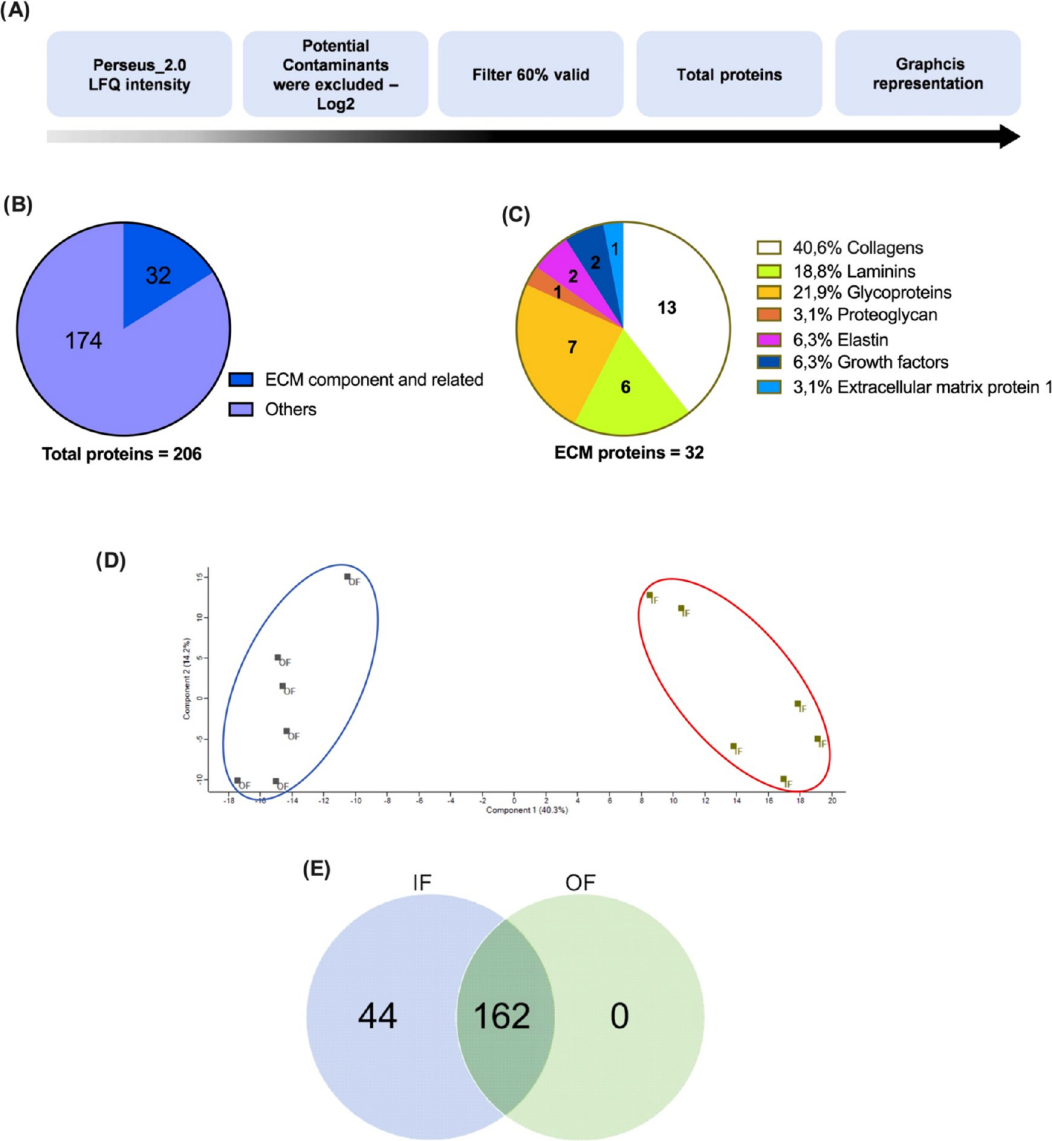
Proteomic analysis of rat adrenal samples and identification of adrenal gland extracellular matrix proteins. (A) Steps of bioinformatic analysis for the obtainment of total proteins identified. (B) Distribution of the 206 designated proteins by ECM-component and ECM-related and others based on the GeneOntology data set (http://geneontology.org). (C) Categorization of the 32 ECM proteins identified. (D) Principal component analysis (PCA) of outer fraction (OF) and inner (IF) fraction sample conditions. (E) Number of proteins common to the fraction samples (162 proteins) and those that were identified only in OF (0 proteins) and in IF (44).

The total number of proteins identified by simultaneously analyzing all of the samples was deposited in the PRIDE repository, project accession PXD046828. They are listed in Table S1. Through the sequential analyses shown in Figure 2A, using a 60% presence filter, a total of 206 proteins were identified from the outer (OF) and inner (IF) fractions (Table S2). Thirty-two (15.5%) proteins of the total were assigned as ECM (Figure 2B). A total of 13 collagens (40.6%), 6 laminins (18.8%), 7 glycoproteins (21.9%), 1 proteoglycan (3.1%), 2 elastin (6.3%), 2 growth factors (6.3%), and the extracellular matrix protein 1 (Ecm1) proteins were identified as ECM-component and ECM-related (Figure 2C). A clear differentiation of the outer (OF) and inner (IF) fraction sample conditions was observed via principal component analysis (PCA), indicating a specific signature for the fraction samples (Figure 2D). A higher abundance of proteins was identified in IF (206 proteins) compared to OF (162) as shown in Table S3 and Figure 2E.

### Adrenal OF and IF ECM Regulation

**Figure 3:**
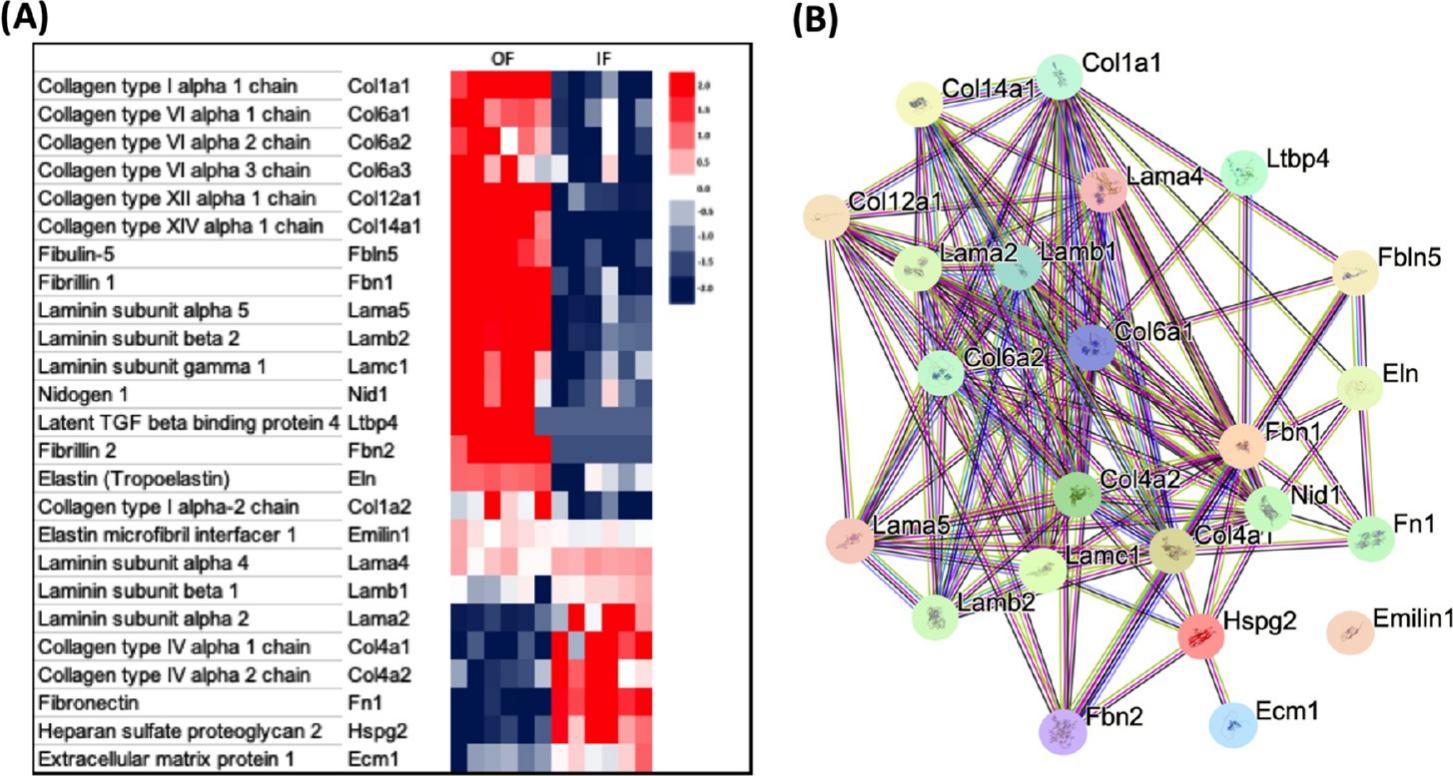
Regulated proteins of the outer and inner fractions of rat adrenal cortex. (A) Heatmap of differentially expressed ECM proteins identified in outer (OF) and inner fractions (IF) of the adrenal cortex. Comparisons by the z-score of the LFQ intensity values, where red and blue represent the higher and lower protein abundance, respectively. (B) Network of protein–protein interaction constructed by the String Consortium tool using Cytoscape software.

A total of 25 proteins out of 32 ECM proteins were significantly regulated (Figure 3A) when the OF and IF fractions were compared. A protein–protein interaction network (https://string-db.org/) for ECM proteins is represented in Figure 3B. The STRING database provides an overview of the network of interactions, which includes direct (physical) and indirect (functional) associations of extracellular matrix proteins. The connections represented by lines and the formation of clusters demonstrate the complexity and number of processes with which these proteins interact. Each process, if considered relevant, can be explored in greater depth in the future.

**Figure 4:**
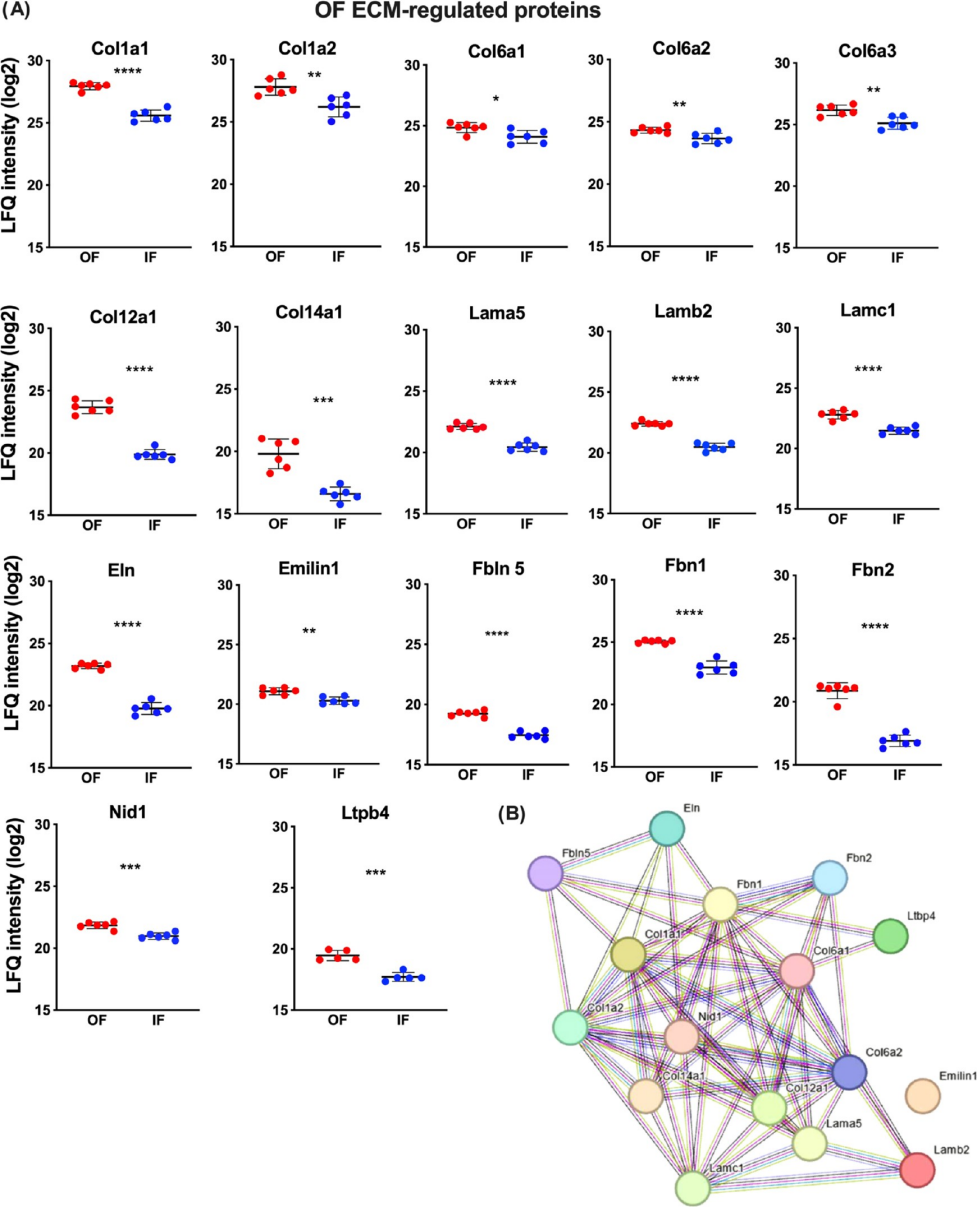
Upregulated ECM proteins in the outer fraction (OF) of the rat adrenal gland. (A) Proteins identified as more abundant in OF (red) by proteomic analysis than those in inner fraction (IF-blue). Student test: *p ≤ 0.05, ** *p* ≤ 0.01, ***p ≤ 0.001, and ****p ≤ 0.0001. The dots represent the LFQ value of each sample, and the horizontal line represents the mean LFQ value ± standard deviation (SD). (B) Protein–protein interaction network constructed by using the String Consortium tool using Cytoscape software.

In OF, 17 ECM proteins are significantly more abundant compared to the inner fraction, 7 collagens (Col1a1, Col1a2, Col6a1, Col6a2, Col6a3, Col12a1, and Col14a1), 3 laminins (Lama5, Lamb2, and Lamc1), 2 elastin (Eln and Emilin), 4 glycoproteins (Fbln5, Fbn1, Fbn2, and Nid1), and the latent TGF β binding protein 4 (Ltbp4) (Figure 4A). A protein–protein interaction network for more abundant ECM proteins in the OF is represented in Figure 4B.

The outer fraction of the adrenal cortex, consisting of the mesenchymal capsule, subcapsule, and zG cells, and the inner fraction consisting of the zF and zR cells, display distinct features. Our study aims to elucidate the influence of ECM on the differences between outer and inner fractions of the rat adrenal cortex through the characterization of the ECM from samples of decellularized adrenal cortex fractions. Our results indicate a certain complexity in the OF adrenal gland, with 17 ECM proteins being more expressed than 8 ECM proteins more abundant in the IF. Considering collagens as the major proteins of the ECM,^18^ our results indicate a signature of Col6a, Col12a1, and Col14a1 in the outer fraction of the adrenal cortex and Col4a1–2 in the inner fraction.

Col6 is made of three different main subunits, a1, a2, and a3 chains, characterized by a relatively short collagenous region and by large globular domains at the N- and C-terminal ends, forming an atypical, beaded microfilament net in the ECM. Col6 plays a mechanical role and cytoprotective function that include inhibition of apoptosis and oxidative damage, the promotion of tumor growth and progression, the regulation of cell differentiation and of autophagic machinery, and the maintenance of cell stemness.^19^ Col12 belongs to the fibril-associated collagen with interrupted triple helices (FACIT) family and is an α1 homotrimer consisting of two short collagenous domains and three noncollagenous domains, including a large N-terminus domain.^20^ There is evidence that Col12 forms ECM complexes that absorb or transduce mechanical signals in response to mechanical stress.^21^ During the regenerative process, Col12 interacts with tenascin, Col1, and fibronectin, where TGF-β signaling is activated. Interestingly, in lesion-specific tissue, the deposition of Col12a1 is regulated by fibroblast-like cells through Wnt/β-catenin signaling, which also regulates the expression of Col6a2 in spinal cord regeneration.^22^ This suggests the possibility of Col12 and Col6 role in the continuous turnover of the adrenal cortex, with steroidogenic cells being born in the outer cortex, followed by centripetal migration and apoptosis in the cortico-medullary boundary as described by Chang and Finco.^23,24^

Col14 is also a member of FACIT.^25^ These collagens are considered incapable of forming collagen fibrils themselves but may interact with collagen fibrils.^26^ Like Col12, Col14 is found in Col1-rich tissues and is known to promote fiber assembly and limit lateral growth.^26,27^ Col14 is prevalent in areas of high mechanical stress, suggesting that Col14 may affect the mechanical properties of tissue.^25^ In the outer fraction of the rat adrenal cortex, Col14 and Col1a are more abundant and coexist in this region, as presented in Figure 4. Previous research has shown that in adult rat adrenal glands, Col1 is the major glycoprotein in the outer cortex, with moderate Col4 levels,^28^ which confirms our observation. However, this is the first time that Col6a1, Col6a2, Col6a3, Col12a1, and Col14a1 have been identified in the rat adrenal cortex.

Morphologically, the outermost zG located beneath the capsule consists of small compact aldosterone-producing cell clusters organized as a rosette-like structure.^29^ In the same region, potential progenitor/stem cells reside within the adrenal capsule or subcapsular space.^3^ Rosette formation is mediated by adherent junctions between epithelial cells that self-organize into a flower-like structure surrounded by a basement membrane (BM) composed of laminin and collagen.^30^ However, the degree of cellular plasticity of ZG is unknown, and the precise nature of zG rosettes, the structure, how and when they arise, and their role in proper zG function have only recently been investigated.^31−34^ Alternatively, one could potentially perform studies introducing Col6a, Col12a, and Col14a to elucidate the importance of the zG structure and function.

In addition to the upregulation of collagens in rat adrenal OF, we identified the upregulation of Fibrillin 1 and 2. Fibrillins constitute a family of extracellular cysteine-rich glycoproteins highly conserved with three homologous members, fibrillin 1, 2, and 3, synthesized and secreted by mesenchymal cells.^35^ Fibrillins form multicomponent structures called microfibrils with 10–12 nm in diameter.^36^ Microfibrils provide tensile strength in nonelastic tissues and act as regulators of growth factor availability and activity in connective tissues.^37^ There is a strongly maintained modular multidomain organization in the fibrillins, with the prevalent epidermal growth factor (EGF)-like domain in fibrillin 2, and a conserved consensus sequence for calcium binding (cbEGF). Calcium binding by cbEGF domains seems to protect the molecule from proteolysis and stabilize the protein for recognition by matrix components.^38−40^ Furthermore, transforming growth factor-β (TGF-β) binding domains can be found in all fibrillin family members.^41^ The binding of TGF-β to fibrillin-rich microfibrils is required for the normal regulation of the local concentration and release of these growth factors, as well as the duration and intensity of the resulting signaling events.^42^ Studies have shown that fibrillin 2 can indirectly bind and sequester TGF-β by interacting with latent TGF-β binding protein (LTBP).^40^ Notably, the latent transforming growth factor β-binding protein 4 (Ltbp4) was observed to be more abundant in OF (Figure 4). LTBP is part of the TGF-β large latent complex, which binds TGF-β in the extracellular matrix.^42^ It was reported that TGF-β signaling inhibits adrenal steroid production by inhibiting StAR, CYP11B1, and CYP11B2.^43,44^ Therefore, TGF-β binding protein Fbn2 may be involved in steroidogenesis in zG by inhibiting TGF-β signaling pathways.

In addition to the aforementioned ECM components, elastin (Eln and Emilin1) and fibulin 5 (Fbnl5) were identified as more abundant. Elastin is an ECM protein that is critical to tissue structural integrity. Cross-linked elastin and associated microfibrils, the elastic fiber, provide the elasticity required for proper tissue function. Fibulins 4 and 5 contribute to the formation of mature elastin by binding to tropoelastin, the precursor to elastin, as well as fibrillins.^45^ Multiple tropoelastin molecules are then cross-linked in desmosine and isodesmosine units to create cross-links by lysyl oxidase (LOX) to generate mature elastic fibers. Mature elastic fibers consist of an insoluble cross-linked elastin center surrounded by microfibrils primarily composed of fibrillin.^46^ As with fibrillin-rich microfibrils, an additional component of mature elastic networks is latent transforming growth factor β binding proteins (LTBPs). LTBP remains within the mature elastic network until released and activated by either mechanical force or proteolytic cleavage.^42^ In elastic fibers, elastin-microfibril-interface-located-protein-1 (EMILIN1) localizes at the interface between the fibrillin microfibril scaffold and the elastin core. The precise role of EMILIN1 in elastogenesis remains unclear.^47^ In vascular smooth muscle cell cultures, pull-down experiments suggest a direct interaction with Eln and Fbln5.^48^ Interestingly, although Emilin1 is more abundant in OF, a protein–protein interaction network for more abundant ECM proteins in OF (Figure 4B) shows no interaction between Emilin and the other OF-identified proteins.

**Figure 5:**
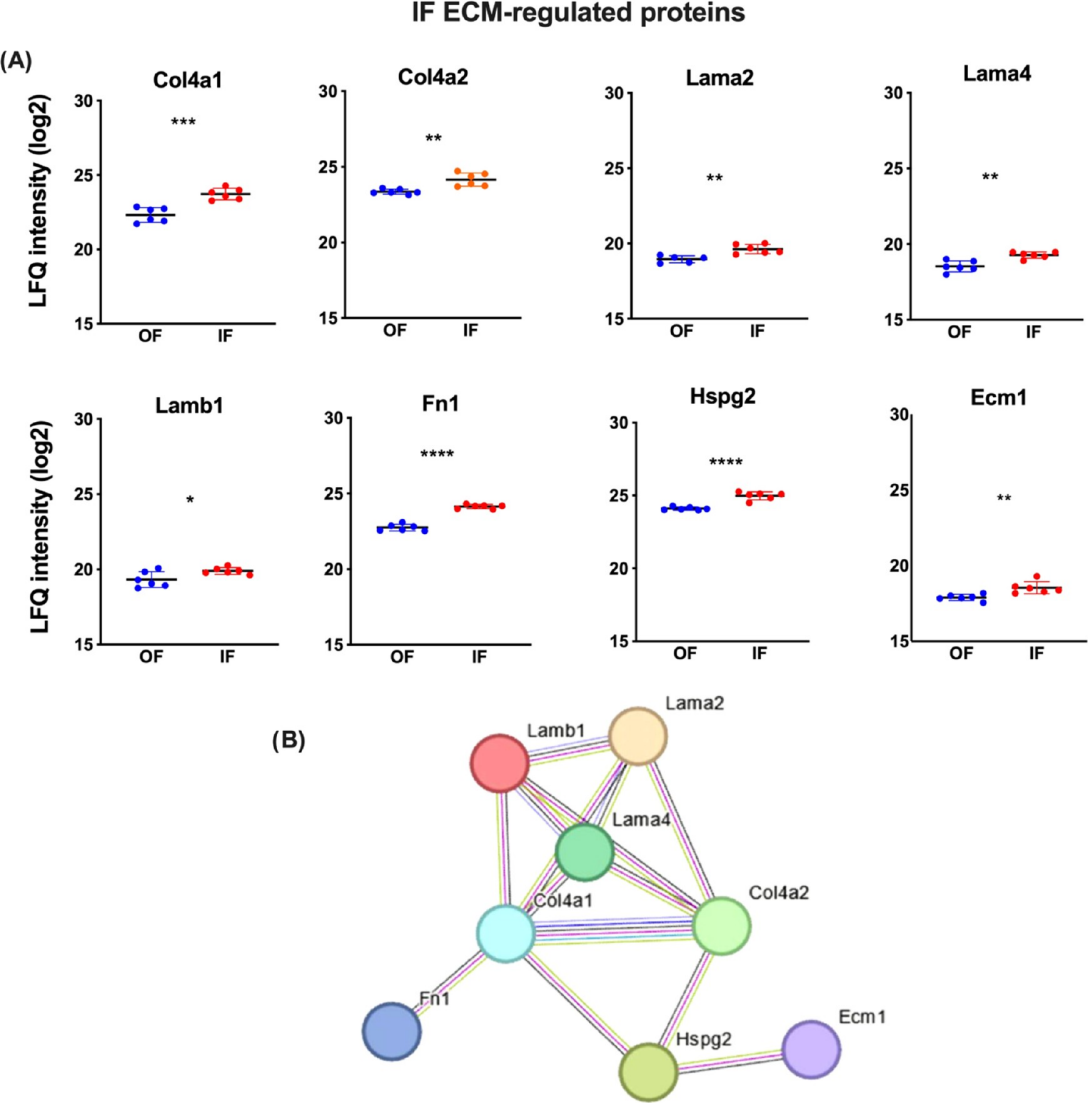
Upregulated ECM proteins in the inner fraction (IF) from rat adrenal cortex. (A) Proteins identified as more abundant in IF (red) by proteomic analysis than those from the outer fraction (OF-blue). Statistical significance was assessed by an unpaired *t* test. *p ≤ 0.05, **p ≤ 0.01, ***p ≤ 0.001, and ****p ≤ 0.0001. The dots represent the LFQ value of each sample, and the horizontal line represents the mean LFQ value ± SD. (B) Protein–protein interaction network constructed by the String Consortium tool using Cytoscape software.

In IF, 8 ECM proteins are significantly more abundant when compared with OF, 2 collagens (Col4a1, Col4a2), 3 laminins (Lama2, Lama4, Lamb1), 1 glycoprotein (Fn1), 1 proteoglycan (Hspg2), and the extracellular matrix protein 1 (Ecm1) (Figure 5A). A protein–protein interaction network for more abundant ECM proteins in IF is represented in Figure 5B. Unlike the Emilin1 protein in OF, which does not interact physically or functionally with any other protein, in OF, all proteins interact.

Equally important for the biological characteristics of the adrenal cortex was the identification of Col4a1 and Col4a2 abundances in the IF of the cortex. Otis and collaborators^9^ examined Col4 in the rat adrenal cortex, which exhibited moderate labeling in the outer adrenal cortex zone, while in zF, Col4 presented long thick fibrils between cords of fasciculata cells, in agreement with our findings in the IF. Col4 promoted ACTH-induced proliferation in both glomerulosa and fasciculata cells in vitro and corticosterone production in fasciculata cells.^9^ Col4 enhanced cell proliferation and favored cortisol secretion after ACTH or Angiotensin II stimulation^49^ in the primary culture of human fetal adrenal cells, together with laminin. In conclusion, identifying more abundance of Col4 in rat adrenal gland IF corroborates the relation of this collagen type to adrenal cortex steroidogenesis and maintenance.

Regarding fibronectin (Fn1) and laminins, we know that in the adult human adrenal gland, Lama2, Lama5, and Lamc1 were found in epithelial BM in all cortical zones, Lama4 in vessels, Lamb1 in the outer zone, and Lamb2 in the two inner zones of the cortex, respectively.^50^ In primary cultures of fetal human adrenal cells, fibronectin (Fn1) and laminin decreased the level of ACTH-stimulated cortisol but enhanced androgen secretion. Concerning cell behavior, laminin enhanced cell proliferation and fibronectin increased cell death.^51^

**Figure 6:**
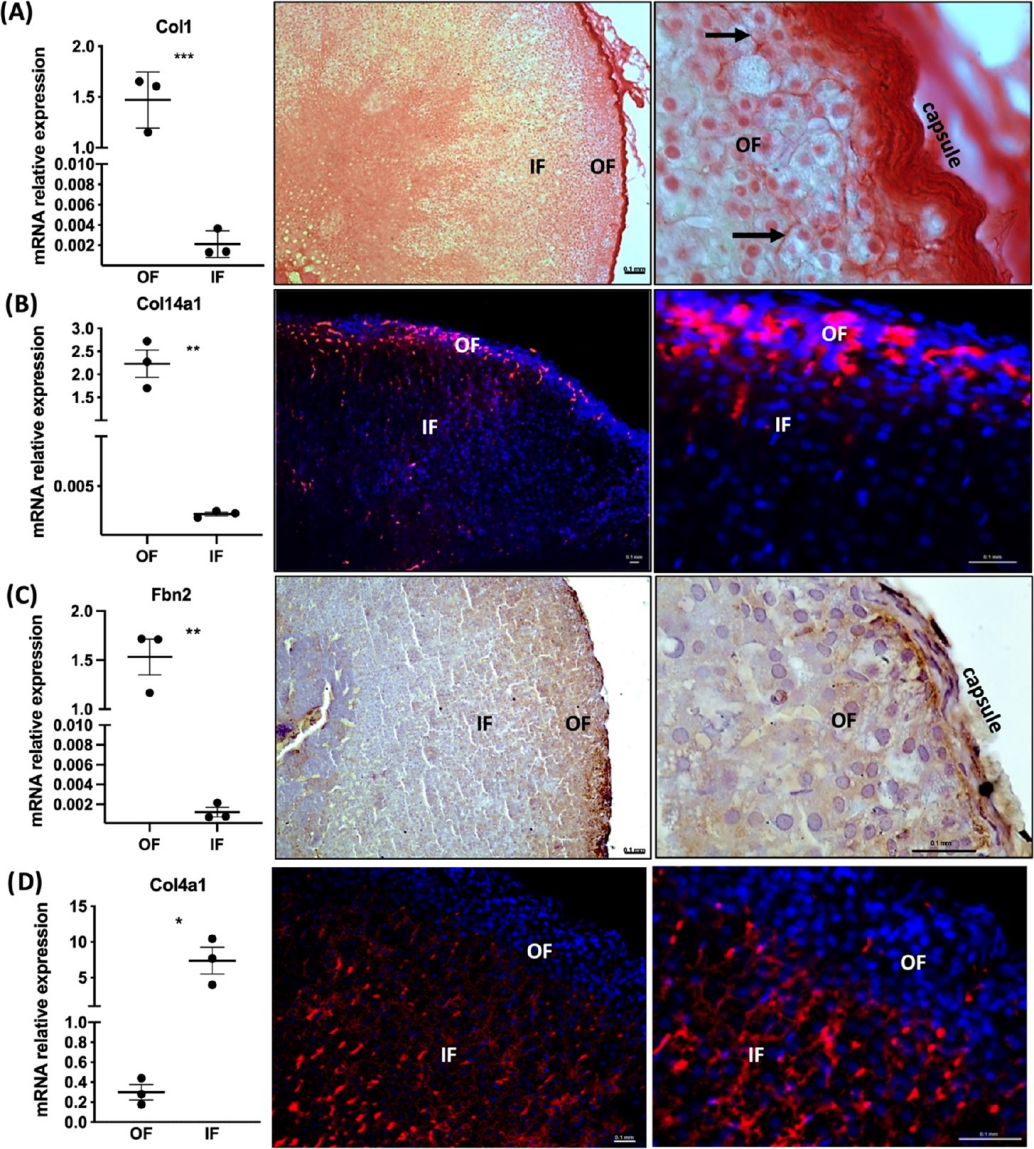
Validation of ECM protein expression in the adrenal outer fraction (OF) and inner fraction (IF). (A) Gene expression analysis and representative images of Picrosirius Red stain for Collagen 1 (Col1). Gene expression and representative images of immunohistochemistry for (B) Collagen 14 (Col14), (C) Fibrillin 2 (Fbn), and (D) Collagen 4 (Col4). Statistical significance was assessed by unpaired *t* test. *p ≤ 0.05, **p ≤ 0.01, ***p ≤ 0.001.

The RT-PCR validation, picrosirius red staining, and immunohistochemistry confirmed the expression of Col1, Col14, and Fbl2 in the OF (Figure 6A–C). Regarding the proteins significantly more abundant in IF than in OF, immunohistochemistry validated Col4a1 expression in IF (Figure 6D).

### ECM Male and Female Composition

**Figure 7:**
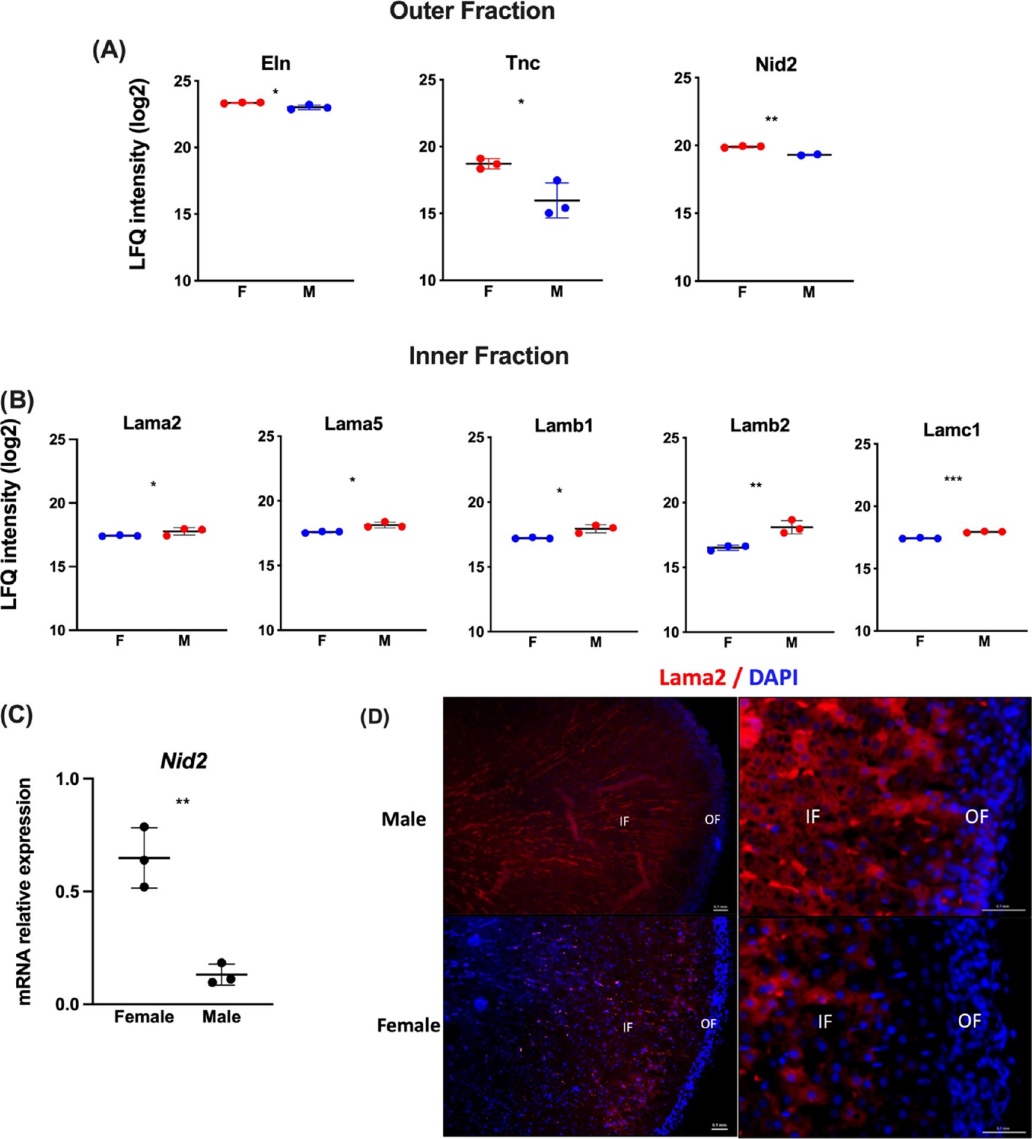
ECM of male and female adrenal cortexes. (A) Proteins were identified as more abundant in the outer fraction of females (F) by proteomic analysis than in males (M). (B) Proteins identified as more abundant in the inner fraction of male IF than those in female IF. Comparisons by the z-score of the LFQ intensity values, where red and blue represent the more and less abundant proteins, respectively. The dots represent the LFQ value of each sample, and the horizontal line represents the mean LFQ value ± SD. (C) Validation of Nid2 gene expression in the OF of male and female samples using qPCR. (D) Validation of Lama2 protein expression in male and female IF samples using immunofluorescence. Statistical significance was assessed by unpaired *t* test. *p ≤ 0.05, **p ≤ 0.01, ***p ≤ 0.001.

The 6 adrenal samples from each fraction, 3 from males and 3 females, and the ECM proteins of males’ and females’ adrenal OF and IF were analyzed. Of the 32 ECM proteins detected, elastin (Eln), tenascin (Tnc), and nidogen-2 (Nid2) proteins were significantly identified as more abundant in the outer adrenal fraction of females (Figure 7A). In the inner fraction of males, 5 laminins (Lama2, Lama5, Lamb1, Lamb2, and Lamc1) presented upregulation (Figure 7B). Validation by RT-PCR and immunohistochemistry confirmed the upregulation of Nid2 gene expression in OF (Figure 7C), and Lama2 protein expression in IF (Figure 7D), respectively.

In rodents, there is notable sexual dimorphism in adrenal gland structure and function, with females displaying higher adrenal weight, plasma ACTH, corticosterone, and aldosterone levels than males.^52,53^ Females had more pronounced activation of the renewal and developmental process of the adrenal cortex, resulting in more diverse adrenal gland structures and function.^54^ For this reason, ECM proteins were investigated from samples of male and female decellularized adrenal cortex fractions. Elastin, (Eln), tenascin (Tcn), and nidogen-2 (Nid2) were identified as more abundant in female OF and different types of laminin chains (Lama2, Lama5, Lamb1, Lamb2, Lamc1) in male IF, respectively (Figure 7). Tenascin was described in the adult adrenal glands of pigs and is considered together with thrombospondins and secreted protein acidic and rich in cysteine belonging to a group of multifunctional extracellular matrix glycoproteins.^55^

Nidogens are sulfated glycoproteins and members of the BM. The BM, a network of insoluble proteins surrounding cells and tissues, comprises laminins, collagen IV, nidogens, and heparan sulfate proteoglycans, functioning as signaling platforms by sequestering many growth factors and other ligands.^56^ Nid2 stabilizes the BM by linking Col4 and laminin to the fibrillar network in the ECM, maintaining cell adhesion by connecting to membrane integrins.^57^ Loss of Nid1 and Nid2 leads to BM ruptures, causing renal agenesis and impaired lung and heart development.^58^ Conversely, Nid1 and Nid2 single mutants show no overt abnormalities, pointing to the redundant role of homologous genes in the assembly and maintenance of BM.^59,60^ Leng^33^ described the presence of rosette-like structures in adult zG across species, which are covered by a BM made up of laminin β1 (Lamb1) and Col4, constituting each glomerular unit within the zG. Given the identification of Nid2 in female OF, we suggest that Nid2 is a constituent of BM in female zG.

Laminins (Ln) are multifunctional proteins recognized by cells by dimeric integrin receptors. They form a protein family of trimeric proteins containing α (a), β (b), and δ (c) chains, forming distinct isoforms in different organ locations, suggesting organ-or tissue-specific functions. Ln has been found in BM through the adrenal cortex and is required for tissue homeostasis.^10,61^ Falk and collaborators^62^ described Lama1 in different BM of mouse adrenal glands; however, in the fetal and adult human adrenal cortex, Lam2 and Lam10 constitute the BM of OF, whereas BM of IF contains Lam4 and Lam11.^50^ Ln is produced by bovine adult adrenal fasciculata cells and has also been shown to promote migration in these cells.^10,61^ In the fetal human adrenal gland, laminin promotes proliferation, prevents apoptosis, and inhibits cortisol production in response to ACTH in vitro.^12,49,51^ However, these findings are unrelated to or specifically related to gender analysis. The presence of Ln plays an important functional role in the adrenal cortex and warrants further study regarding the relationship between Lama2 and the IF of male rats.

As recognized limitations of the study design, we can point out the pooled samples, which, despite impacting sample variability, were grouped for ethical reasons to limit the number of animals sacrificed. Furthermore, the approach was considered to increase the number of protein identifications. Another point is the decellularization protocol. Even with meticulous decellularization protocols, cellular remnants that remain after decellularization may interfere with proteomic analysis by contributing to background noise or masking low-abundance proteins. The decellularization process can lead to the loss of certain proteins, modification of the proteomic profile description, and accurate assessment of the protein composition of the tissue. Also, during decellularization, the proteins may undergo modifications altering the protein structure and function, making it challenging to identify and characterize them accurately via proteomic analysis. Regarding proteomic techniques, they may present limited sensitivity to detect low-abundance proteins. Also, it may not be broad enough to accurately quantify proteins with abundances of several orders of magnitude in abundance. The analysis of proteomic data from decellularized tissues is complex due to the volume of data generated. Integration of proteomics data with other data sets, such as transcriptomics, may be necessary to gain a comprehensive understanding of tissue composition and function.

## Conclusions

**Figure 8:**
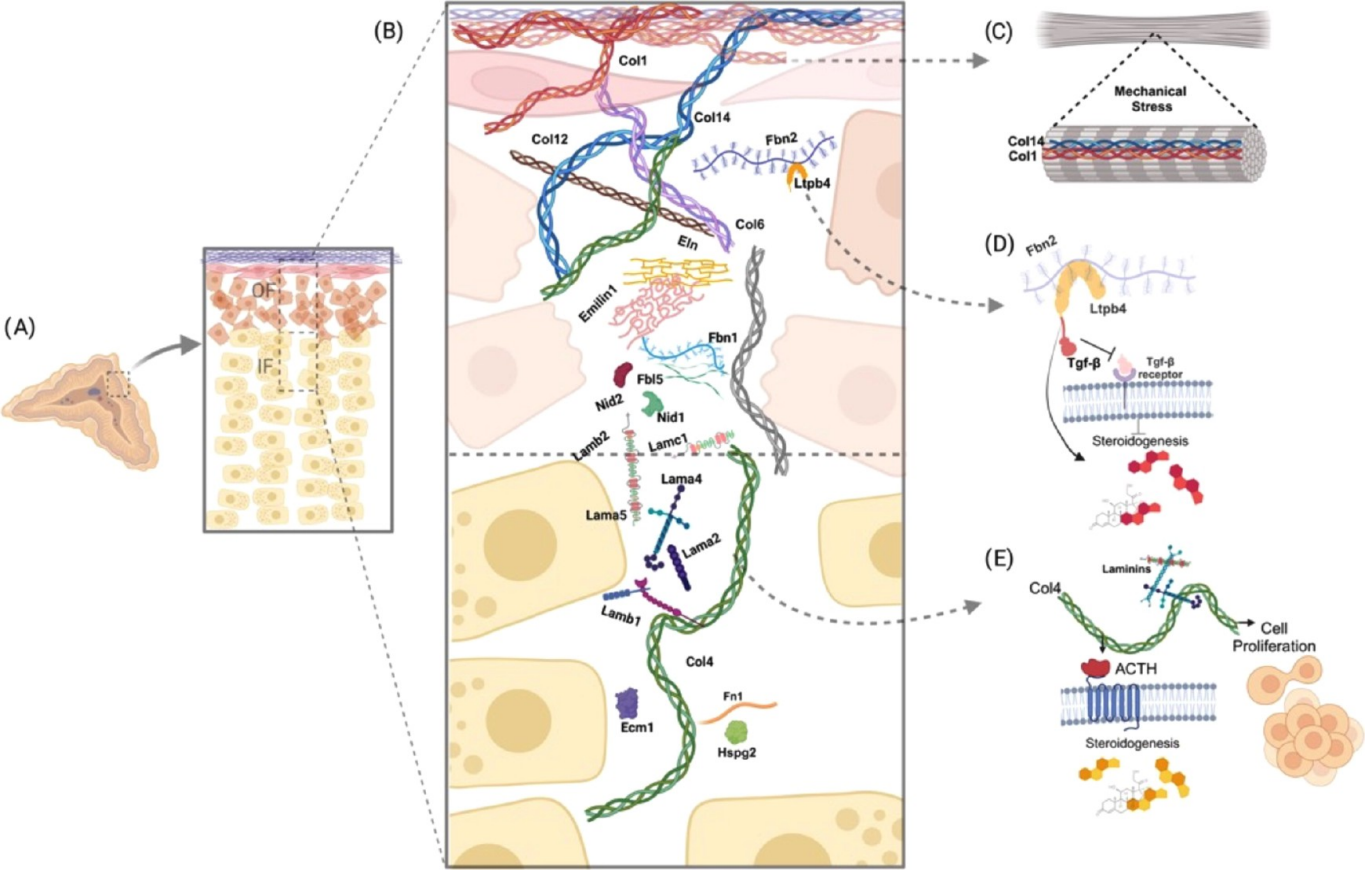
Illustrative model representing the most abundant proteins in the rat adrenal cortex. (**A**) The representation of the rat adrenal gland with cortex and medulla, and the subdivision of the cortex into an outer (OF cells of the capsule, subcapsular, and glomerulosa) and an inner fraction (cells of the fasciculata). (B) Expansion of OF and IF to observe elements of the extracellular matrix (ECM) identified as collagens, laminins, nidogens, and others. (C) Col14 can interact with Collagen Type 1 (Col1) for the assembly of strength fibers in regions subject to tension. (D) Fbn2 may act as an indirect regulator for transforming growth factor-β (TGF-β) through its interaction with Ltpb4, which in turn may be involved in steroidogenesis by modulating TGF-β signaling pathways. (E) In IF, particular attention is given to the identification and upregulation of Collagen Type 4 (Col4), which, when associated with laminins, seems to be related to ACTH-induced cell proliferation and steroidogenesis in vitro. The remaining proteins identified as being more abundant were represented in the respective fractions, and their specific functions require further investigation. Created with BioRender.com.

Our study represents the first comparative analysis of the ECM-specific proteome of outer and inner fractions of the rat adrenal cortex. The components and the related ECM proteins of the outer and inner fractions of the adrenal cortex demonstrate their distinct compositions (Figure 8). In the outer fraction, a rich and complex protein signature suggests tissue stress, stiffness areas, and regulatory proteins for growth factor signaling. In the inner fraction, the upregulation of Col4a1 and Col4a2 and its role in steroidogenesis should be further investigated. Nid2 and Lam2 proteins were also detected in the ECM of female OF and male IF, respectively, both components of BM. How significant the gender differences are needs to be further investigated. Moreover, the other ECM proteins that were identified will be a database for future investigations.

## Supporting Information Available

Certification of decellularization of adrenal fraction samples (Figure S1) (PDF)
Total number of proteins identified in all of the samples (Table S1) (XLSX)
Total protein identified in decellularized fractions of rat adrenal gland (Table S2) (XLSX)
Proteins identified in decellularized inner and outer fractions of rat adrenal gland (Table S3) (XLSX)
