# Supplementary material for: Extracellular Matrix Protein Signatures of the Outer and Inner Zones of the Rat Adrenal Cortex
Source: J Proteome Res. 2024 Jul 17;23(8):3418–32. doi: 10.1021/acs.jproteome.4c00071 (PMC11301687; doi:10.1021/acs.jproteome.4c00071)
Supplement: Supplementary file 1 — pr4c00071_si_002.pdf [file pr4c00071_si_002.pdf]

A)

DNA quantification ng/µL		
Sample	**OF**	**IF**
Control	471,5 ± 33,9	547,3 ± 55,2
Decellularized	42,4 ± 6,1	35,8 ± 3,4

OF - outer fraction; IF - inner fraction

B)

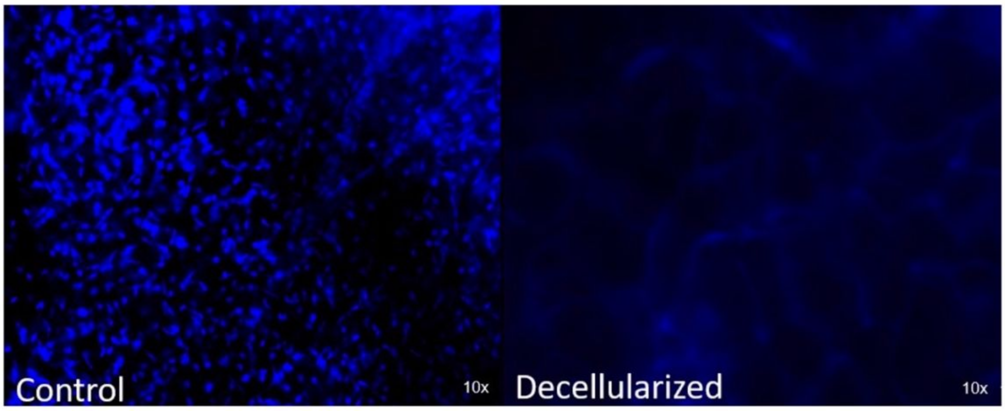

Figure S1 – Certification of decellularization of adrenal fraction samples. A) DNA quantification; B) staining with DAPI (4′,6-diamidino-2-phenylindole).
